# Wavelet Entropy-Based Inter-subject Associative Cortical Source Localization for Sensorimotor BCI

**DOI:** 10.3389/fninf.2019.00047

**Published:** 2019-07-23

**Authors:** Simanto Saha, Md. Shakhawat Hossain, Khawza Ahmed, Raqibul Mostafa, Leontios Hadjileontiadis, Ahsan Khandoker, Mathias Baumert

**Affiliations:** ^1^School of Electrical and Electronic Engineering, The University of Adelaide, Adelaide, SA, Australia; ^2^Department of Electrical and Electronic Engineering, United International University, Dhaka, Bangladesh; ^3^Department of Electrical and Computer Engineering, Khalifa University of Science and Technology, Technology and Research, Abu Dhabi, United Arab Emirates; ^4^Department of Electrical and Computer Engineering, Aristotle University of Thessaloniki, Thessaloniki, Greece; ^5^Healthcare Engineering Innovation Center (HEIC), Department of Biomedical Engineering, Khalifa University of Science and Technology, Abu Dhabi, United Arab Emirates; ^6^Electrical and Electronic Engineering Department, University of Melbourne, Parkville, VIC, Australia

**Keywords:** inter-subject sensorimotor dynamics, brain computer interface, wavelet based maximum entropy on the mean, motor imagery, electroencephalography

## Abstract

We propose event-related cortical sources estimation from subject-independent electroencephalography (EEG) recordings for motor imagery brain computer interface (BCI). By using wavelet-based maximum entropy on the mean (wMEM), task-specific EEG channels are selected to predict right hand and right foot sensorimotor tasks, employing common spatial pattern (CSP) and regularized common spatial pattern (RCSP). EEG from five healthy individuals (Dataset IVa, BCI Competition III) were evaluated by a cross-subject paradigm. Prediction performance was evaluated via a two-layer feed-forward neural network, where the classifier was trained and tested by data from two subjects independently. On average, the overall mean prediction accuracies obtained using all 118 channels are (55.98±6.53) and (71.20±5.32) in cases of CSP and RCSP, respectively, which are slightly lower than the accuracies obtained using only the selected channels, i.e., (58.95±6.90) and (71.41±6.65), respectively. The highest mean prediction accuracy achieved for a specific subject pair by using selected EEG channels was on average (90.36±5.59) and outperformed that achieved by using all available channels (86.07 ± 10.71). Spatially projected cortical sources approximated using wMEM may be useful for capturing inter-subject associative sensorimotor brain dynamics and pave the way toward an enhanced subject-independent BCI.

## 1. Introduction

Most brain computer interfaces (BCI) require subject-specific training sessions, which can annoy users and limit BCI applications such as affective states assessment (Andujar et al., 2015), lie detection (Wang et al., 2016), and gaming (van de Laar et al., 2013). Furthermore, not all the users are able to control BCI due to BCI illiteracy (Allison and Neuper, 2010) and spatio-temporally complex resting state network (RSN) dynamics over time and across individuals (Jensen et al., 2011). Many factors including time-variant psychophysiological, neuroanatomical traits, and user's basic characteristics essentially cause unreliable estimate of RSN, which engender short and long-term brain signal variation over time and across individuals (Goncalves et al., 2006; Ahn and Jun, 2015; Kasahara et al., 2015; Zhang et al., 2015; Acqualagna et al., 2016; Athanasiou et al., 2017). Resting state electroencephalography (EEG)-derived spectral entropy and power spectral density are associated with sensorimotor BCI performance (Zhang et al., 2015; Acqualagna et al., 2016). Attention and motivation are psychological predictors that reflect sensorimotor BCI performance (Hammer et al., 2012). Taking anatomical information such as electrode positioning and head morphologies into consideration can augment subject-to-subject transfer learning and thus BCI performance (Wronkiewicz et al., 2015).

The inter-subject and inter-session variabilities of brain dynamics significantly degrade the performance of EEG-based BCI (Saha et al., 2018). Subjects who show dissociative brain responses, i.e., responses with negligible commonalities across individuals, cannot be accommodated by a generic BCI framework. On the contrary, subjects showing significant commonalities in their brain responses achieve relatively high performance in the context of subject independent motor imagery (MI) classification (Saha et al., 2016, 2018). A recent EEG-based experiment on drowsiness detection signifies the influences of intra- and inter-subject variability and has proposed multi-subject transfer framework for reducing calibration time (Wei et al., 2018).

In this paper, we explore inter-subject associative brain responses to identify pairs of individuals who demonstrate similar EEG dynamics during MI tasks. We anticipate higher classification accuracy in people with higher inter-subject associativity. Previous works used the Indian Buffet process and Kullback-Leibler divergence to identify inter-subject associative individuals prior to classifying multi-subject EEG signals and showed that inter-subject associativity could be potentially used for multi-subject subspace learning (Kang et al., 2009; Kang and Choi, 2014). Thus, inter-subject associative BCI could eliminate the need for subject-specific calibrations despite differences in cortical activities across subjects, mostly because of various time variant psychophysiological factors (Goncalves et al., 2006) and individuals' basic characteristics (Ahn and Jun, 2015). Compensating for inter-subject difference in brain responses can be crucial for improving BCI performance. One approach is to select inter-subject associative EEG channels that exhibit robust activation during specific cortical tasks (Saha et al., 2016). The implicit assumption is that eliminated channels represent RSN and subject-specific MI dynamics free from signatures that are common between subjects. Moreover, individual brain dynamics sometimes show inter-subject cortical association during external stimulation, i.e., visual (Hasson et al., 2004) and auditory (Abrams et al., 2013) events. Subjects having common psychological perspective share associative brain responses during natural vision (Lahnakoski et al., 2014). An ensemble of classifiers has been used to classify mental states from single trial inter-subject EEG recordings (Fazli et al., 2009). In carefully selected subjects, common spatial pattern (CSP)-based subspaces learning methods deal with inter-subject/session data efficiently (Samek et al., 2013, 2014). Subject independent BCI is currently feasible (Abibullaev et al., 2013) and could be used in research, rehabilitation, and gaming (Rana et al., 2013). A recent study used a particle swarm optimization based inter-subject common feature learning technique for BCI implementation without subject-specific training (Atyabi et al., 2017). An unsupervised spectral transfer using information geometry has shown promising classification accuracy in cases, using fewer or no trials from the target subject (Waytowich et al., 2016).

The aim of this study is to identify inter-subject associative electromagnetic sources, estimated from wavelet-based maximum entropy of the mean (wMEM) of single trial EEG, and project them into a three-dimensional (3D) head model. We show that wMEM captures associative inter-subject sensorimotor dynamics, which can be utilized to assess inter-subject cortical associativity.

## 2. Methods

### 2.1. Data and Experimental Settings

**Figure 1:**
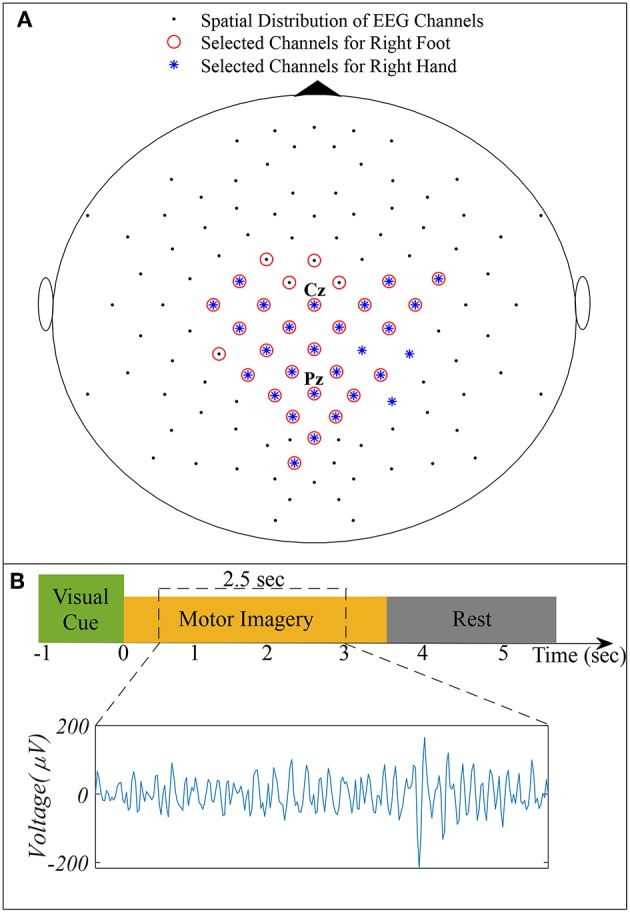
**(A)** Spatial distribution of 118 EEG channels based on the extended International 10/20 System and selected channels for subject pair *al* − *ay*. **(B)** Timing of the recording paradigm for dataset IVa of BCI Competition III and example of selected 2.5 s EEG signal for channel *Cz*.

We used dataset IVa of BCI Competition III, comprising EEG of five healthy subjects specified as *aa, al, av, aw*, and *ay* recorded during right hand and right foot MI (Blankertz et al., 2006). The dataset consists of 280 trials for each subject, i.e., 140 trials for each class. A visual cue was given before each trial, consisting of 3.5 s of EEG recordings with 118 electrodes (Extended 10/20 system). To eliminate the effect of visual cues, 2.5 s of recordings following 0.5 s of the visual cues were considered for experimentation. Figure 1 illustrates the spatial distribution of EEG channels (Extended 10/20 System) and the timing of recording paradigm.

**Figure 2:**
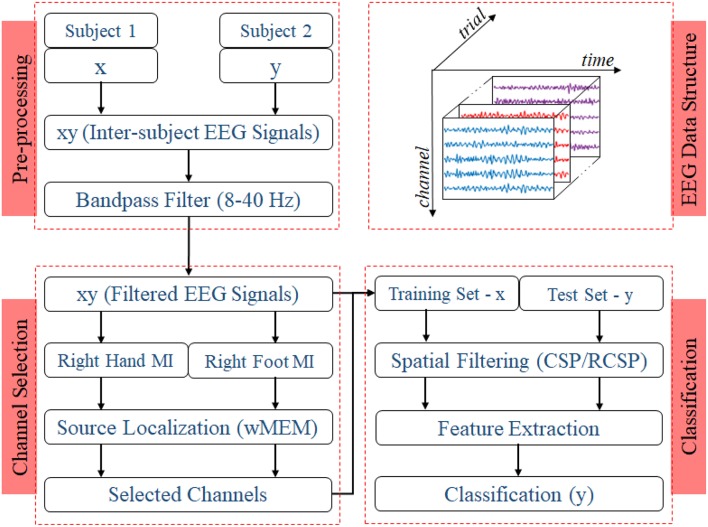
Block diagram representing the EEG trial structure and the proposed methodology to identify inter-subject associative EEG channels and to evaluate the BCI performance. Preprocessing step includes constructing a set of EEG trials from two different subjects with a ratio of 1:1 and applying a Bandpass filter with corner frequencies of 8 and 40 Hz. Inter-subject trials are separated according to the nature of motor imagery tasks, i.e., right hand or right foot. Class-specific sets of trials were used to estimate inter-subject cortical sources and, consequently, EEG channels. For evaluating the BCI performance, trials from one subject were used to establish the single-trial BCI classifier model, which was then evaluated on the trials acquired from a different subject.

Two experiments for evaluating the inter-subject associative sources and estimating the inter-subject classification performance, respectively, were carried out on any two subjects at once as shown in Figure 2, i.e., all possible pairs of the subjects set were considered. Then, we compared the classification performance achieved by using all 118 channels versus that achieved by the selected channels.

Any two subjects' EEG from the set of five participants (*aa, al, av, aw*, and *ay*) are represented as *X* and *Y*, respectively. The total number of trials *Tr* available for each subject (280) was divided into 10 equal sets as follows:

(1) $$\begin{array}{r} X=\left[x_{1},x_{2},\ldots \ldots \ldots ,x_{10}\right];Y=\left[y_{1},y_{2},\ldots \ldots \ldots ,y_{10}\right]. \end{array}$$

Then, sets of inter-subject EEG are derived:

(2) $$\begin{array}{r} XY=\left[x_{1}y_{1},x_{2}y_{2},\ldots \ldots \ldots ,x_{10}y_{10}\right], \end{array}$$

where each component of *XY* contains $\frac{Tr}{10}$ trials from subject *X* that were used to train the classifier and the remaining $\frac{Tr}{10}$ trials from subject *Y* that were used to test the classifier. On each set of *XY*, wMEM was computed to estimate inter-subject associative cortical sources and to test the classification performance. The classification performance was averaged over the 10 sets.

Figure 2 shows the block diagram demonstrating the experimental settings corresponding to preprocessing, channel selection, and classification of inter-subject EEG signals. In the preprocessing step, inter-subject set of EEG trials were band-pass filtered (8 and 40 Hz), using a Butterworth filter of order 10 (Saha et al., 2016, 2018). For each class, trials were separated to apply wMEM method to investigate class-specific inter-subject associative cortical sources and then to select EEG channels representing the mostly activated sensorimotor sources. The union of two channel sets corresponding to right hand and right foot was considered the set of optimal channel set for specific subject pair. The numbers of channels used in this study varied between two different cases: *Case I*—all available 118 channels were employed for classification; *Case II*—only those channels selected by the wMEM approach (<118) were employed for classification. Apart from the numbers of channel, all other parameters were kept identical for evaluating BCI classification performance.

To evaluate inter-subject BCI performance, common spatial patterns (CSP) without and with covariance estimation regularization (Ramoser et al., 2000; Lotte and Guan, 2011) were computed to spatially project multichannel EEG. Wavelet decomposition-based subband entropy (Daubechies 3, level: 3) was calculated to obtain features (Saha et al., 2016, 2018) for a two-layer feed-forward neural network classifier as described previously (Svozil et al., 1997).

### 2.2. Wavelet-Based Source Localization

#### 2.2.1. The Time-Frequency Forward Model and EEG Source Distribution

Assuming that oscillatory brain activities result from underlying processes occurring at different frequency bands located in extended cortical areas (Lina et al., 2014), a *time-frequency (t-f)* forward model utilizing discrete wavelet transform of the data as well as the brain sources along with spatial clustering in homogeneous parcels can be defined (Tadel et al., 2011). To calculate the discrete wavelet transform, real Daubechies filter banks with four vanishing moments were used. The complete details of the numerical implementation of wMEM method can be found in Lina et al. (2014). In this study, we assume that single trial inter-subject EEG signals result from diverse physiological background activity and MI induced cortical activities. Ensemble-averaging over class-specific MI trials effectively reduces the effect of independent physiological fluctuations and, thus offers a potential tool for locating sensorimotor cortical sources. The underlying hypothesis is that wMEM based cortical sources on inter-subject EEG trials set would give only inter-subject associative cortical sources, in which only the inter-subject common EEG patterns related to right hand or right foot MI are detected.

The *t-f* forward model depends on the lead field matrix that governs the relationship between co-registered bioelectric sources and set of sensors. A generalized anatomical MRI template (ICBM152) estimated from MRI scans acquired from 152 healthy subjects, was used to create a realistic head model (Fonov et al., 2011). The template is included in the Brainstorm software (Tadel et al., 2011). The template comprises three head layers, i.e., scalp, outer skull, and inner skull, which were approximated using T1 MRI sequences. It exhibits good contrast and captures fine definition of the outermost boundary of the brain. OpenMEEG software was then used to solve steady-state Maxwell equations to calculate the lead field matrix by establishing a realistic relationship between bioelectric sources in the ICBM152 and co-registered sensors on the scalp (Kybic et al., 2006; Gramfort et al., 2010).

The EEG inverse solution utilizes a distributed source model, where a large number of dipolar sources are disseminated across the cortex. Depending on the anatomical restrictions, each dipole is oriented orthogonal to the local cortical surface. The linear relation of the source amplitude to the recordings can be written as

(3) $$\begin{array}{r} \text{M}=\text{G J}+\text{E}, \end{array}$$

where **M** is the recording matrix of size (*q* × τ), which contains EEG signals of *q* channels at τ time samples. ***E*** represents Gaussian recording noise. ***J*** is an unknown matrix of the size (*r* × τ) that represents the current density of the *r* dipolar sources along the tessellated cortical surface. ***G*** represents the lead field matrix (*q* × *r*). ***G*** is estimated by solving the *t-f* forward problem that evaluates the contributions of all dipolar sources to the electrodes. Hence, the inverse solution approximates ***J*** from the recorded data ***M*** and the evaluated lead field matrix ***G***.

#### 2.2.2. wMEM Inverse Solution

Solving the ill-posed inverse problem of source localization requires some *a priori* information to be incorporated in the regularization framework to obtain a unique solution. In the *MEM* framework, the amplitude of the sources **J** is considered a multivariate random variable *j* of *r* dimensions that has a probability distribution *dp*(*j*). In the *MEM* framework, to regularize the inverse problem some previous knowledge on *j* is incorporated as reference distribution *dv*(*j*). This reference distribution represents a realistic spatial model which presumes the brain activity is organized into *K*(*K* < < *r*) cortical parcels and every parcel is related to a secret state variable. This variable controls the parcel activity, whether or not the parcel is active during a certain task. A data-driven parcellization method is used to spatially cluster the cortical surface into *K* non-overlapping parcels. The technique consists of first applying a projection method named as the multivariate source pre-localization technique, estimating a probability-like coefficient for every dipolar sources distributed along the cortical mesh. This coefficient characterizes the contribution of every source to the data, followed by region growing around local maxima.

In the *MEM* reference model, every parcel is assigned a secret variable to model the probability of the parcel's activity, whether or not it is active. It is to be noted that the multivariate source pre-localization coefficients of all the sources within the parcel was used to initialize this probability. Based on the state of activation of the parcels, *MEM* inference can switch any of these parcels on or off. It is also able to approximate a contrast of source intensities within the selected active parcels. Usually, *MEM* is applied for solving the inverse problem in the time domain. *wMEM* is the wavelet variant of MEM that operates in the *time-frequency* (Lina et al., 2014).

While the joint probability of the wavelet coefficient of all sources at a specific time and scale is represented as *p*(*w*), the *MEM* estimation deduces the expectation *E*_*p*_[*w*] by assuming a reference probability μ(*w*) from which the entropy deviation is minimized under the goodness-of-fit data constraint. The entropy *S*_μ_(*f*) of any μ density, *p*(*w*) = *f*(*w*)μ(*w*), can be described as follows:

(4) $$\begin{array}{r} S_{\mu }\left(f\right)=-\int_{w}f\left(w\right)\text{ln}f\left(w\right)\mu \left(w\right)dw, \end{array}$$

here *w* are the wavelet coefficients of the sources and $w^{*}=E_{p^{*}}\left[w\right]$ will give the optimum solution, where p^*^(*w*) = f^*^(*w*)μ(*w*), considering

(5) $$\{\begin{array}{c} f^{*}={\text{argmax}}_{f}S_{\mu }(f).\\ \text{with}\int_{w}Gwf(w)\mu (w)dw=d^{*}, \end{array}$$

where d^*^ can be obtained from $d^{*}=W^{-1}\delta_{\tau }\left(w\right)$ and $W=\overset{-\frac{1}{2}}{\underset{d}{\sum }}$ is the whitening matrix. This whitening matrix can be evaluated from a baseline EEG recording assuming no signal of interest is contained in the baseline recording. δ_τ_(*w*) represents the soft shrinkage of the wavelet coefficient which can be defined as

$$\begin{array}{l} \delta_{\tau }\left(w\right)={\left(1-\frac{\tau }{\left|w\right|}\right)}_{+}w \end{array}$$

where *w*_+_ = *w* for *w* > 0 and *w*_+_ = 0 for other cases. The threshold τ of each channel is obtained from the variance of the highest frequency wavelet coefficients, evaluated using a median estimator.

With respect to optimum μ-density f^*^, the mean of the wavelet coefficients of the sources is used to estimate the expectation of the coefficient. Finally, the expectation of the coefficient with respect to the f^*^, can be expressed as the *a posteriori* mean estimate of the wavelet coefficients:

(6) $$w^{*}={\int wf^{*}(w)\mu (w)dw=\frac{d}{d\xi }F_{\mu }^{*}(\xi )|}_{\xi =G^{t}\lambda^{*}},$$

with $F_{\mu }^{*}\left(\xi \right)=\text{ln}\int e^{\xi^{t}w}\mu \left(w\right)dw$ and $\text{ln}Z\left(\lambda \right)=F_{\mu }^{*}\left(G^{t}\lambda \right)$. In (6) λ^*^ is the unique solution of

$$\lambda ={\text{argmax}}_{\lambda }D\left(\lambda \right),$$

where

$$D\left(\lambda \right)=\lambda^{t}d^{*}-F_{\mu }^{*}\left(G^{t}\lambda \right)-\frac{1}{2}\lambda^{t}\eta^{*}\lambda$$

and η^*^ is the covariance matrix of residual noise.

For localizing cortical sources, either the time course of the sources obtained by inverse wavelet transform or spatial cortical map of the wavelet coefficients have been considered. A complete description of wMEM-based localization can be found in Lina et al. (2014).

To implement the wMEM-based source localization for the inter-subject EEG, the open source Brainstorm software was used (Tadel et al., 2011). MI induced sources were projected into the 3D anatomical head model. For each class, all available trials were averaged and a noise covariance matrix was estimated. Finally, the *BrainEntropy MEM* with the *wMEM* option was used to calculate the sources from the averaged data.

#### 2.2.3. Inter-subject Associative EEG Channel Selection

**Figure 3:**
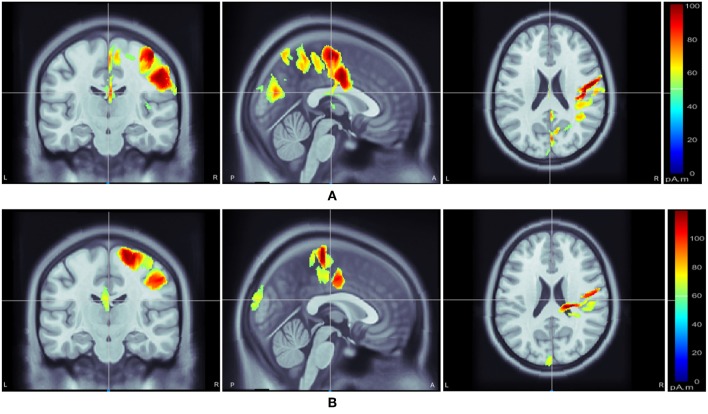
Motor imagery induced inter-subject (subject pair *al-ay*) cortical sources on a MRI head model estimated via wMEM: from left-right, coronal view, sagittal view, and axial view, respectively for two motor imagery tasks: **(A)** right hand and **(B)** right foot movement.

**Figure 4:**
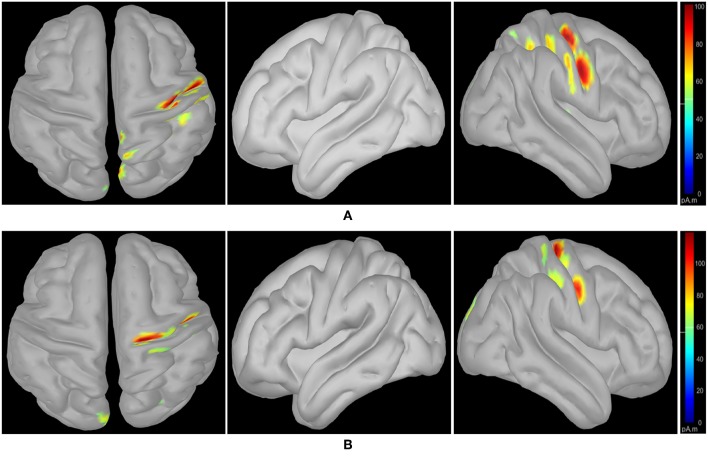
wMEM based inter-subject (subject pair *al-ay*) associative cortical source localization illustrated on a 3D cortex cartoon model: from left-right, top view, side view (right hemisphere), and side view (left hemisphere), respectively for **(A)** right hand and **(B)** right foot motor imagery. In **(B)**, the activity visible in the top view is not clearly projected on the side view (left hemisphere) as the activated sources lie mostly within relatively inner parts of the cortex (in between gyri).

Following class-specific wMEM-based cortical source localization, careful visual inspection on the MRI head images (i.e., coronal, sagittal, and axial view) and 3D cortex cartoon model as shown in Figures 3, 4, respectively, was carried out. For each class, all time instances were examined for activated sources within the trial duration, i.e., 0–2.5 s. The EEG channels located on/around the estimated sources on the 3D head model template were considered as task-specific inter-subject associative optimal channels. Figures 3, 4 illustrate inter-subject associative cortical source estimation for subject pair *al-ay* at a time instance. Notably, the 3D cortex head model can be examined visually by rotating the view at any of the 360^0^.

### 2.3. Common Spatial Pattern With and Without Covariance Estimation Regularization

The aim of CSP is to maximize the difference between class specific features (Ramoser et al., 2000). Here, we use CSP with tuning the covariance estimation, which effectively deals with undesired outliers and is suitable in cases of small training trials (Lu et al., 2010; Lotte and Guan, 2011).

The EEG signal is represented by *E* and of size *N* × *P*, where *N* is the number of channels and *P* is the number of samples per trial. For the conventional CSP algorithm, the sample based covariance matrix estimation is required. The sample covariance matrix of a trial *E* is normalized to the total variance as (Ramoser et al., 2000; Lu et al., 2010).

(7) $$\begin{array}{r} \text{S}=\frac{\text{E}{\text{E}}^{T}}{\text{trace }\left[{\text{EE}}^{T}\right]}, \end{array}$$

where ^*T*^ denotes the transpose of a matrix.

If *K* trials are available for training corresponding to each class for a subject, indexed by *k* as **E**_(c,k)_ that refer to the **S**_(c,k)_, based on (7), *k* = 1, 2, ……, *K*, the mean sample covariance matrix across the trials is given by

(8) $$\begin{array}{r} {\bar{\text{S}}}_{c}=\frac{1}{K}\overset{K}{\underset{k=1}{\sum }}{\text{S}}_{\left(\text{c},\text{k}\right)}, \end{array}$$

where *c* ∈ {1, 2} represents two classes of the trial associated with the MI tasks.

The discriminative spatial patterns in CSP are calculated based on the sample mean covariance matrix estimation based on (8). The next section will introduce regularization in CSP.

#### 2.3.1. Covariance Matrix Estimation With Regularization

Regularization is achieved by biasing the covariance estimation away from their sample-based values toward more physically plausible values, which reduces the variance of the sample-based estimates while tending to increase bias (Lu et al., 2010). This is done by using one or more regularization parameters (i.e., β and γ in this paper).

The regularized average spatial covariance matrix for each class is defined as

(9) $${\hat{\sum }}_{c}(\beta ,\gamma )=(1-\gamma ){\hat{\Omega }}_{c}(\beta )+\frac{\gamma }{N}\text{trace}\left[{\hat{\Omega }}_{c}(\beta )\right]\cdot \text{I}.$$

Here, β and γ are regularization parameters (0 ≤ β, γ ≤ 1) and *I* is the identity matrix. ${\hat{\Omega }}_{c}\left(\beta \right)$ comprises the covariance matrix for the trials from the specific subjects, as well as generic trials, and is given by

(10) $$\begin{array}{r} {\hat{\Omega }}_{c}\left(\beta \right)=\frac{\left(1-\beta \right).{\text{S}}_{c}+\beta .{\hat{\text{S}}}_{c}}{\left(1-\beta \right).M+\beta .\hat{M}}. \end{array}$$

Here, **S**_*c*_ is the sum of the sample covariance matrices for all *M* training trials for class *c* :

(11) $$\begin{array}{r} {\text{S}}_{c}=\overset{K}{\underset{k=1}{\sum }}{\text{S}}_{\left(\text{c},\text{k}\right)}. \end{array}$$

${\hat{\text{S}}}_{c}$ is the sum of the sample covariance matrices for $\hat{K}$ generic training trials with covariance matrix ${\text{E}}_{\left(c,\hat{k}\right)}$ for class *c*:

(12) $$\begin{array}{r} {\hat{\text{S}}}_{c}=\overset{\hat{K}}{\underset{\hat{k}=1}{\sum }}{\text{S}}_{\left(\text{c},\hat{\text{k}}\right)}. \end{array}$$

Here, **S**_*c*_ and ${\hat{\text{S}}}_{c}$ are normalized and are analogous to the sample covariance matrix in (7). The objective of ${\hat{\text{S}}}_{c}$ is to reduce the variance in the covariance matrix estimation and produce more reliable results.

#### 2.3.2. Feature Extraction in RCSP

The regularized composite spatial covariance is formed and factorized as Lu et al. (2010)

(13) $$\begin{array}{r} \hat{\sum }\left(\beta ,\gamma \right)={\hat{\sum }}_{1}\left(\beta ,\gamma \right)+{\hat{\sum }}_{2}\left(\beta ,\gamma \right)=\hat{\text{U}}\hat{\wedge }{\hat{\text{U}}}^{T}. \end{array}$$

Here, $\hat{\text{U}}$ denotes the matrix eigenvectors and $\hat{\wedge }$ denotes the diagonal matrix of corresponding eigenvalues. The eigenvalues are assumed to be sorted in descending order throughout this paper (Ramoser et al., 2000; Lu et al., 2010).

Finally, the projection matrix is formed as Lu et al. (2010)

(14) $$\begin{array}{r} \hat{\text{W}}={\hat{\text{B}}}^{T}{\hat{\wedge }}^{-1/2}{\hat{\text{U}}}^{T}, \end{array}$$

where $\hat{\text{B}}$ denotes the matrix of eigenvectors for the whitened spatial covariance matrix and defined as

(15) $$\begin{array}{r} \hat{\text{B}}={\hat{\wedge }}^{-1/2}{\hat{\text{U}}}^{T}{\hat{\sum }}_{c}\left(\beta ,\gamma \right)\hat{\text{U}}{\hat{\wedge }}^{-1/2}. \end{array}$$

In RCSP, an input trial E is projected as Lu et al. (2010)

(16) $$\begin{array}{r} \hat{\text{X}}={\hat{\text{W}}}^{T}\text{E}. \end{array}$$

To obtain the most discriminative features for both classes, the optimal channels are to be selected from the leftmost and rightmost channels. For example, the first channel represents the most distinguished features for class 1 and the last channel represents the most distinguished features for class 2. As the channel selection converges to the central channel of the $\hat{\text{X}}$, the features become poor and may hardly distinguish different classes. It is to be noted that RCSP equals traditional CSP when β = γ = 0.

The following combinations of γ and β values were considered during regularization (Wang et al., 2006; Lu et al., 2010; Saha et al., 2018):

β = (0, 0.001, 0.01, 0.1, 0.2, 0.3, 0.4, 0.5, 0.6, 0.7, 0.8, 0.9)

γ = (0, 0.01, 0.1, 0.2, 0.3, 0.4, 0.5, 0.6, 0.7, 0.8, 0.9).

A total of four CSP components, two for each class, were selected for extracting features (Wang et al., 2006; Sannelli et al., 2016).

## 3. Results and Discussion

### 3.1. Selection of Inter-subject Associative Channels

Figures 3, 4 illustrate inter-subject associative source locations for subject pair *al-ay* at a time instance. Table 1 lists the total number of selected channels used to classify MIs (*Case II*). Although the number of selected channels differed between right hand and right foot MI, many common channels were identified for both classes. Some of the projected cortical sources lie within deeper regions of the brain, thus, maintaining good signal-to-noise ratio in the scalp EEG becomes critical. Modeling the signal attenuation from cortical sources located within deeper brain areas to the EEG electrode montages might play an important role for dealing with noise efficiently (Cosandier-Rimélé et al., 2008). Specific parts of the interconnected cortico-subcortical networks show sensorimotor signatures. For example, basal ganglia and the ventrolateral part of the thalamus show sensorimotor activations (Gerardin et al., 2000; Hétu et al., 2013). Projections of sensorimotor activities to deeper brain areas are present in motor cortex and supplementary area (Hétu et al., 2013). Possibly, sensorimotor activities in subcortical networks manifest in EEG signals. Previous works have exploited subject-specific EEG source localization based information for improving sensorimotor BCI performance (Congedo et al., 2006; He et al., 2015). The cortical sources corresponding to MI shown in Figures 3, 4, lie mostly within subcortical areas. The validation of the implication about the subcortical sources as mostly activated during inter-subject MI require further investigation. Notably, it has been hypothesized that the estimated sources delineate only the inter-subject associative sources; the MI sources that are not common in the subject pair should not present in this experimental context, because, the source localization method was applied on inter-subject set of EEG comprising an equal number of trials from each subject.

**Table 1:** Number of selected EEG channels.

**Subject pair**	**RH**	**RF**	**Total**
aa-al	36	36	43
aa-av	33	39	42
aa-aw	40	42	44
aa-ay	42	37	45
al-av	36	37	43
al-aw	40	23	46
al-ay	28	30	33
av-aw	42	37	46
av-ay	47	45	55
aw-ay	46	54	59

*RH, Right Hand; RF, Right Foot*.

### 3.2. Motor Imagery Prediction Performance

Table 2 shows the MI prediction performance averaged over ten sets of inter-subject data of two subjects. Each set consisted of 56 trials in total, the first 28 trials from a subject were used to model the classifier and the remaining 28 trials from another subject were used to evaluate the performance. Each subject pair was used twice, alternating the training and evaluation subjects. The highest prediction performance (90.36±5.59%) was achieved for subject pair *ay-al* using CSP with covariance regularization. This demonstrates the feasibility of inter-subject BCI for subjects who show similar EEG patterns. In this case, only 33 of the 118 available channels were employed, reflecting the inter-subject associative cortical areas. Alternating the order of training and evaluation trials for subject pair *al-ay* reduced the prediction performance to (84.64±13.15%), suggesting that the performance of CSP depends on the training data. Since CSP is a data driven method it can be overfitted, adapting to outliers in the training set (Sannelli et al., 2016). Tuning of the covariance estimation via two regularization parameters, γ and β can alleviate this problem. Overall, regularization of the covariance matrix enhanced prediction performances compared to standard CSP. For subject pair *aa-al*, significant improvements in performances were evident (64.64±14.33 vs. 76.79±9.11%), further demonstrating the potential of wMEM as a tool for localizing inter-subject associative cortical sources.

**Table 2:** Single trial motor imagery prediction performances.

	**CSP**		**RCSP**	
**Subject**	**Case I**	**Case II**	**Case I**	**Case II**
**pair**	**Mean ± SD**	**Mean ± SD**	**Mean ± SD**	**Mean ± SD**
aa-al	51.79 ± 14.70	64.64 ± 14.33	73.21 ± 9.71	76.79 ± 9.11
aa-av	54.64 ± 10.25	55.71 ± 7.38	66.79 ± 6.53	66.07 ± 10.00
aa-aw	55.36 ± 10.28	62.86 ± 11.93	68.93 ± 10.92	72.86 ± 7.93
aa-ay	53.93 ± 12.76	58.93 ± 17.11	75.00 ± 8.58	72.14 ± 13.24
al-av	53.93 ± 6.40	51.07 ± 4.47	68.21 ± 6.40	65.71 ± 7.75
al-aw	62.50 ± 7.39	53.93 ± 11.22	72.50 ± 11.91	71.79 ± 10.44
al-ay	73.57 ± 13.38	71.78 ± 11.47	83.21 ± 12.26	84.64 ± 13.15
av-aw	49.29 ± 8.55	53.93 ± 14.43	70.36 ± 6.74	68.57 ± 9.34
av-ay	62.50 ± 10.81	62.86 ± 11.81	72.50 ± 10.39	71.79 ± 8.49
aw-ay	47.50 ± 10.39	53.21 ± 11.84	66.79 ± 7.54	70.00 ± 9.25
al-aa	56.79 ± 10.97	58.93 ± 11.82	72.50 ± 5.84	70.71 ± 13.55
av-aa	52.14 ± 13.70	52.50 ± 5.84	67.14 ± 9.34	64.29 ± 7.14
aw-aa	56.07 ± 9.68	56.43 ± 13.86	64.64 ± 7.23	63.21 ± 8.43
ay-aa	57.86 ± 9.49	56.07 ± 10.11	69.29 ± 6.56	70.36 ± 11.05
av-al	46.07 ± 8.82	55.36 ± 12.17	71.79 ± 7.80	71.79 ± 9.74
aw-al	63.93 ± 14.72	68.21 ± 20.86	69.64 ± 12.05	75.71 ± 15.50
ay-al	63.21 ± 20.55	77.14 ± 14.40	86.07 ± 10.71	**90.36** **±** **5.59**
aw-av	54.29 ± 11.88	55.36 ± 5.89	66.43 ± 5.38	65.71 ± 4.52
ay-av	53.93 ± 8.82	54.29 ± 5.53	68.93 ± 8.08	66.07 ± 4.84
ay-aw	50.36 ± 10.02	55.71 ± 5.11	70.00 ± 6.12	69.64 ± 5.39
Mean(Mean) ± SD(Mean)	55.98 ± 6.53	58.95 ± 6.90	71.20 ± 5.32	71.41 ± 6.65

*CSP, Common Spatial Pattern; RCSP, Regularized Common Spatial Pattern; Case I, all channels; Case II, selected channels. The meaning of bold values are highest prediction performance*.

**Figure 5:**
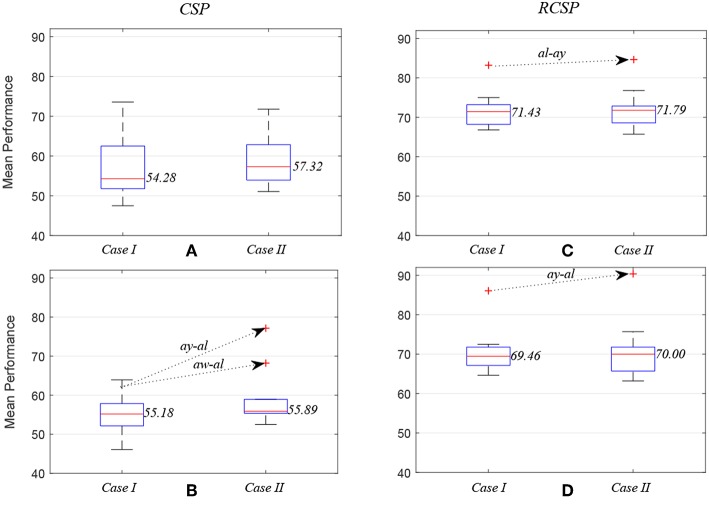
Box plots illustrating mean prediction performances for different subject pairs: the performances were measured while applying common spatial pattern **(C,D)** with (RCSP) and **(A,B)** without (CSP) covariance estimation regularization.

Figure 5 compares average prediction performance and highlights cases where selected EEG channels outperformed results obtained when using all available 118 channels. In Figures 5A,C, compare the mean performances of first ten subject pairs summarized in Table 2, while Figures 5B,D compare the mean performances of the last 10 subject pairs for CSP without and with covariance regularization, respectively. Applying CSP with covariance estimation, the median values of average classification accuracies for the first 10 subject pairs using all channels and selected channels are 71.43% and 71.79%, respectively, while they were 69.46% and 70% for last 10 subject pairs. Thus, the overall results might not indicate a generalized trend of improved performance of inter-subject BCI performance. However, particular subject pair-specific (*al*−*ay*) improved performance would suggest a novel application of wMEM and a probable role in investigating inter-subject sensorimotor dynamics. A reduced number of channels can be used to achieve comparable performance while lessening the computational cost.

### 3.3. Enhanced Inter-subject Associative Sensorimotor Dynamics

Localizing task induced cortical sources is important because specific sources provide more informative and inter-class distinguishable attributes for predicting sensorimotor events. The minimum current estimates algorithm, applied to magnetoencephalogram recordings, suggest that the contralateral motor cortex is highly active during intended movement direction (Wang et al., 2010). Estimating sensorimotor cortical sources with fine spatial resolution EEG source imaging could augment BCI in decoding complex MI tasks (Edelman et al., 2016). Measurement of entropy by different means was shown to be useful for cortical source localization. For example, Von Neumann entropy was applied to classify MI tasks (Kamousi et al., 2007) and wavelet ridge analysis-based MEM was applied to localize EEG sources (Zerouali et al., 2013). However, the cortical sources vary widely in the spatiotemporal domain across subjects making the inter-subject source localization challenging, as evident from an electromagnetic spatiotemporal independent component analysis-based multi-subject EEG study (Tsai et al., 2014).

Selecting inter-subject associative cortical sources can not only be used to identify optimal task induced EEG channels, but can also provide enhanced weight of associativity between subjects. Not well-understood variability due to functionally relevant RSN (Wens et al., 2014) and, sometimes outliers (Arvaneh et al., 2011) might manifest in undesired channels, which negatively contribute to the prediction performance. Basically, efficiently dealing with the EEG inverse problem is challenging as the solution is non-unique and unstable (Grech et al., 2008). However, the results presented here suggest that wMEM is a potential tool for approximating cortical sources originating inside the cortico-subcortical networks. Improved prediction accuracies with a reduced number of selected inter-subject channels indicate enhanced associativity of the subjects' sensorimotor dynamics. In Hossain et al. (2016), wMEM was adopted as a channel selection tool in subject-specific BCI settings for the first instance. Clarke and Jandey proposed MEM for solving the inverse problem (Clarke and Janday, 1989) and Rice had proposed this method as the most probable solution to the inverse problem in EEG considering realistic neurophysiological constraints (Rice, 1990). Lina et al. have recently proposed wMEM in the context of localizing epileptic sources from EEG data (Lina et al., 2014).

### 3.4. Study Significance and Limitation

Subject-to-subject and session-to-session transferability of trained model parameters is inevitably critical for the generalization of a BCI system (Jayaram et al., 2016). In supervised machine learning-based applications, a principal assumption is that the training and test data follow similar distributions, which often fails and, consequently covariate shift occurs (Pan and Yang, 2010). Covariate shift adaptation has been a key strategy to compensate for inter-subject and inter-session variability in BCI (Sugiyama et al., 2007). However, this study investigated inter-subject associativity in cross-subject BCI paradigm (Saha et al., 2016, 2018). The aim was to evaluate if there are similarities between two subjects' neural substrates quantified by wMEM-based inter-subject cortical source localization for MI tasks. Exploiting inter-subject associativity, i.e., leveraging source space related neuroscience priors may augment transfer learning (Wronkiewicz et al., 2015) and reduce/eliminate the calibration effort for BCI. The advantage of our cross-subject paradigm over a pooled-subject paradigm is that it allows to directly assess associativity of any two subjects' brain dynamics in source space. We suggest that wMEM selection of EEG channels could advance this goal beyond the reduction in computational cost due to fewer analyzed channels because it has been hypothesized that the selected channels manifest the inter-subject associative sensorimotor dynamics in source space.

It is to be noted that our results do not indicate a common trend of BCI prediction performance for all subject pairs. Our goal was to identify pairs of subjects sharing common sensorimotor dynamics. Thus, achieving poor BCI prediction performance for any subject pair might manifest dissimilar MI-related dynamics between subjects (Saha et al., 2016, 2018). On the other hand, it might not be improbable to achieve good prediction performance for one subject pair only, assuming both subjects share common sensorimotor dynamics related to right hand and right foot MI. Further studies would be necessary to explore the role of wMEM-based cortical source estimation in subject pairs showing poor classification performance. This study is limited by using data from a few subjects with no individual information on head/brain anatomy.

Another key limitation of this study is the manual selection of EEG channels by visually inspecting the MI-related source activation projected on the 3D head geometry. To the best of our knowledge, this study is the first attempt to investigate the role of any source localization method on inter-subject EEG signals on BCI context to evaluate inter-subject associativity. Future studies should aim at extracting optimal channels automatically, by imposing selection criteria in the 3D head model geometry source space.

## 4. Conclusion

Brain dynamics reflected on RSN are complex and variable across individuals. Thus, compensating for inter-subject variability is important for calibration-free BCI. In this paper, we have demonstrated that wMEM could be used to identify inter-subject associative sources within the cortico-subcortical networks, which allow selecting optimal EEG channels for classifying subject independent MI tasks. The improved prediction performance utilizing fewer, optimal EEG channels results in enhanced inter-subject coherence and suggests the suitability of wMEM for assessing inter-subject associative sensorimotor oscillations.

## Author Contributions

SS conceived the idea of investigating inter-subject sensorimotor associativity using wMEM, designed the experimental paradigm, and wrote the first draft. SS and MH implemented wMEM and generated all the results. KA, RM, LH, AK, and MB participated in the interpretation of the results and provided critical feedback on the writing. All authors read the final manuscript.

### Conflict of Interest Statement

The authors declare that the research was conducted in the absence of any commercial or financial relationships that could be construed as a potential conflict of interest.
